# The Olive Phenolic *S*–(–)–Oleocanthal as a Novel Intervention for Neuroendocrine Prostate Cancers: Therapeutic and Molecular Insights

**DOI:** 10.3390/nu17243947

**Published:** 2025-12-17

**Authors:** Md Towhidul Islam Tarun, Hassan Y. Ebrahim, Dalal Dawud, Zakaria Y. Abd Elmageed, Eva Corey, Khalid A. El Sayed

**Affiliations:** 1 School of Basic Pharmaceutical and Toxicological Sciences, College of Pharmacy, University of Louisiana at Monroe, 1800 Bienville Drive, Monroe, LA 71201, USA; tarunmt@warhawks.ulm.edu (M.T.I.T.); hebrahim@ulm.vcom.edu (H.Y.E.); dawuddr@warhawks.ulm.edu (D.D.); zelmageed@ulm.vcom.edu (Z.Y.A.E.); 2 Department of Biomedical Sciences, Discipline of Pharmacology, Edward Via College of Osteopathic Medicine, Monroe, LA 71203, USA; 3 Department of Urology, University of Washington, Seattle, WA 98195, USA; ecorey@uw.edu

**Keywords:** de novo and treatment-induced neuroendocrine prostate cancer, *S*–(–)–oleocanthal, LuCaP 93 Patient-Derived Xenograft, recurrence, RNA sequencing, ROR2-ASCL1-SMYD2-EZH2-c-MET axis

## Abstract

**Background/Objectives**. Prostate cancer (PCa) is among the leading causes of death from cancer in men. Frequent use of androgen receptor inhibitors induces PCa transdifferentiation, leading to poorly differentiated neuroendocrine PCa (NEPC). ROR2 is critical for NEPC pathogenesis by activating ASCL1, promoting lineage plasticity. Protein lysine methylation mediated by *N*-lysine methyltransferases SMYD2 and its downstream effector EZH2 upregulates the NEPC marker ASCL1 and enhances c-MET signaling, promoting PCa aggression. Epidemiological studies suggest a lower incidence of certain malignancies in Mediterranean populations due to their intake of an olive-phenolics-rich diet. **Methods**. Cell viability, gene knockdown, and immunoblotting were used for in vitro analyses. A nude mouse NEPC xenograft model evaluated the anti-tumor efficacy of purified and crude oleocanthal. Xenograft tumors were subjected to RNA-seq, qPCR, and Western blot analyses, with clinical validation performed using tissue microarrays. **Results**. A tissue microarray analysis showed that SMYD2 expression was significantly elevated in PCa tissues with higher IHS versus normal prostate tissue cores. The olive phenolic *S*–(–)–oleocanthal (OC) suppressed the de novo NEPC NCI-H660 cells proliferation. Male athymic nude mice xenografted with the NCI-H660-Luc cells were used to assess OC effects on de novo NEPC progression and recurrence. Male NSG mice transplanted with LuCaP 93 PDX tumor tissues generated a heterogeneous in vivo model used to assess OC effects against t-NEPC progression. Daily oral 10 mg/kg OC administration significantly suppressed the NCI-H660-Luc tumor progression and locoregional recurrence after primary tumor surgical excision. OC treatments effectively suppressed the progression of LuCaP 93 PDX tumors. OC-treated tumors revealed downregulation of ROR2, ASCL1, SMYD2, and EZH2, as well as activated c-MET levels versus the placebo control. RNA sequencing of the collected treated NEPC tumors showed that OC disrupted NEPC splicing, translation, growth factor signaling, and neuronal differentiation. **Conclusions**. This study’s findings validate OC as a novel lead entity for NEPC management by targeting the ROR2-ASCL1-SMYD2-EZH2-c-MET axis.

## 1. Introduction

Prostate cancer (PCa) is the most prevalent cancer among men in the U.S., and the second leading cause of cancer-related death. About 313,780 new PCa cases will be diagnosed in 2025, and 35,770 men are expected to die of PCa [1,2]. PCa predominantly depends on androgen signaling for survival and growth, making androgen deprivation therapy, combined with androgen receptor (AR)-targeting agents such as abiraterone acetate or enzalutamide, the standard first-line treatment for metastatic PCa [3]. Approximately 20% of CRPC cases transdifferentiate to AR-independent neuroendocrine prostate cancer (NEPC), posing significant diagnostic and therapeutic challenges [4,5]. NEPC manifests either as de novo, averaging 1–2% of PCa cases, or the more common treatment-induced NEPC (t-NEPC), which develops following prolonged AR-targeted therapies [6,7]. Treatment options for NEPC are limited, including platins, etoposide, or taxanes [8]. However, these interventions achieve modest therapeutic benefits, and their use is associated with high toxicity and uncontrolled disease progression [9].

The atypical receptor tyrosine kinase (RTK)-like orphan receptor 2 (ROR2) regulates multiple biological processes, including embryonic development, epithelial–mesenchymal transition (EMT), invasions, and phenotype switching [10]. ROR2 is aberrantly overexpressed in NEPC cell lines and patient samples compared to primary castration-sensitive PCa (CSPC) [10,11]. ROR2 has also been shown to enhance ENZ-induced plasticity by regulating the lineage commitment achaete–scute family bHLH transcription factor 1 (ASCL1) [10,11]. ROR2 exhibits tumor suppressor activity in colon cancer and hepatocellular carcinoma driven by canonical Wnt signaling [10].

The protein lysine methyltransferases SMYD family regulate cellular lysine methylation through their SET and MYND domains. SMYD2 methylates histones and non-histone proteins (EZH2 and others), modulating key cellular processes [12]. The SMYD2 gene is frequently amplified in colon, pancreatic, lung, triple-negative breast, and prostate cancers [13,14,15,16,17]. SMYD2 methylates EZH2 at lysine 307, altering protein bulkiness, hydrophobicity, and interactions with methyl-lysine readers, activating downstream pathways [14,18]. EZH2 overexpression is prevalent in cancers, including NEPC, reinforcing its role in malignancy [19]. The RTK c-MET drives tumor invasiveness and metastasis, including advanced PCa [20]. c-MET is dysregulated in NEPC and associated with therapy resistance [20].

Epidemiologically, consumption of extra-virgin olive oil (EVOO)-rich Mediterranean diets is associated with a reduced incidence of various cancers, including PCa [21]. The key bioactive EVOO phenolic ingredient, *S*–(–)–oleocanthal (OC), has shown anticancer properties by targeting multiple oncogenic mediators [22]. In one study, OC competitively inhibited c-MET kinase phosphorylation, reducing cancer cell proliferation, invasion, and angiogenesis, controlling c-MET-driven tumor progression [23]. OC has also been shown to suppress breast cancer progression and recurrence, emphasizing its potential as a therapeutic nutraceutical [24,25]. Another study showed that OC suppressed the metastatic CRPC (mCRPC) progression and recurrence by direct inhibition of SMYD2, with a favorable safety profile [26]. OC has also been shown to significantly modulate SMYD2, EZH2, and c-MET in colorectal cancer [27].

There are no current in vivo reports on the NEPC-suppressive effects of any bioactive natural product. Thus, OC is hypothesized to control NEPC progression and recurrence by targeting distinct oncogenic pathways. This study reports OC’s in vivo anti-NEPC effects, along with molecular mechanistic insights. The results highlight OC potential as a novel lead intervention for effective NEPC management.

## 2. Materials and Methods

### 2.1. Chemicals and Reagents

Most chemicals used in this study were purchased from Avantor Science Central (Allentown, PA, USA), unless otherwise stated. *S*–(–)–oleocanthal (OC) was extracted from a Greek EVOO (The Governor, Corfu, Greece) using a liquid–liquid extraction method, followed by entrapment on SP70 resin [28]. OC-rich fraction was then eluted with acetone after water displacement. Final OC purification was performed using Sephadex LH20 chromatography with CH_2_Cl_2_ and gradient increasing EtOAc amounts as the mobile phase. A ≥99% OC purity was achieved and confirmed by q^1^H NMR analysis [28]. OC stock solution was prepared as described earlier, and stored in amber, airtight glass vials at −20 °C, protected from light and humidity, and thawed only once immediately prior to dosing [25,26,27,28,29].

### 2.2. Cell Lines and Culture Conditions

The human de novo NEPC NCI-H660 cells were obtained from the American Type Culture Collection (ATCC, Rockville, MD, USA, Supplementary Table S1). Cells were cultured in ATCC-formulated RPMI-1640 medium supplemented with 0.005 mg/mL insulin, 0.01 mg/mL transferrin, 30 nM sodium selenite, 10 nM hydrocortisone, 10 nM 17β-estradiol, an additional 2 mM L-glutamine (resulting in a final concentration of 4 mM), and 5% fetal bovine serum (FBS). Cultures were maintained at 37 °C in a humidified atmosphere containing 5% CO_2_. Cell density was routinely monitored, and the medium was replenished every 2–3 days to sustain optimal growth conditions. For subculturing, flasks were gently agitated to dislodge aggregated cells, since the viable cells typically formed in clusters. Subculturing was performed when multiple healthy clusters were observed to ensure continued cell proliferation and viability.

### 2.3. NCI-H660 Cell Transfection

Cells were seeded into 12-well plates and cultured until reaching 60–70% confluency. A master mix was prepared for transfection by adding 0.5 µg SMYD2-specific shRNA plasmid (Santa Cruz Biotechnology, Dallas, TX, USA) to Lipofectamine 2000 reagent (Thermo Fisher Scientific, Carlsbad, CA, USA) with 100 µL of Opti-MEM reduced serum media (Gibco, Gaithersburg, MD, USA). This mixture was incubated at room temperature (rt) for 20 min. The culture media were then aspirated, and cells were gently washed with PBS. Then, 100 µL of the prepared Opti-MEM mixture (with or without the Lipofectamine/plasmid complex) was added to each well, and cells were incubated for 4–6 h at 37 °C. The transfection media were then aspirated and replaced with complete serum-containing medium, and cells were further incubated for 48 h. Transfection efficiency was assessed by Western blot analysis to confirm the SMYD2 knockdown (Supplementary Table S2).

### 2.4. Cell Viability Assay

The NCI-H660 cells were seeded at a density of 5000 cells/well in a 96-well plate containing different OC treatments with complete RPMI-1640 medium containing 5% FBS and incubated for 5 days at 37 °C with 5% CO_2_. About 20 µL of MTS reagent was added to each well, and the plate was incubated for 3–4 h to allow color development. Absorbance was measured at 490 nm using a microplate reader (BioTek, Winooski, VT, USA). Absorbance values were normalized to the control wells, and data analyzed to assess the effects of OC treatments versus vehicle control.

### 2.5. Lentivirus-Aided Luciferase Labeling of NCI-H660 Cells

NCI-H660 cells were seeded into 12-well plates and cultured until reaching 60–70% confluency. Lentiviral particles carrying the luciferase gene (Kerafast, Boston, MA, USA) were added to cells. The lentivirus vector was prepared by mixing Opti-MEM reduced serum media (1.5 µL/100 µL) and gently mixing on ice. After aspirating the culture media, cells were washed with PBS, and 100 µL of Opti-MEM media—with or without viral particles—was added to each well. The cells were incubated for 4–6 h at 37 °C, after which the transfection media were replaced with complete serum-containing medium for further culture over a few days. To select luciferase-expressing cells, puromycin (15 µg/mL, Santa Cruz Biotechnology, Dallas, TX, USA) was added to the culture media. The puromycin-containing media were replaced every 2 days to ensure the elimination of non-transduced cells. Luciferase activity was assessed by adding 20 µL of 50 mM XenoLight D-luciferin K^+^ salt bioluminescent substrate (PerkinElmer, Waltham, MA, USA) in PBS to each well, followed by incubation for 6 min at rt. Bioluminescence was confirmed using PerkinElmer’s IVIS imaging platform, ensuring successful luciferase tagging.

### 2.6. Tissue Microarray Immunohistochemistry and Histochemical Score

A paraffin-embedded tissue microarray (TMA) slide containing 192 tissue cores from 64 human cases—58 PCa specimens and 6 normal or hyperplastic prostate tissues—was procured from US Biomax (Derwood, MD, USA) with the associated clinical data. Prior to analyzing the TMA, antibody optimization was carried out using three distinct PCa tissue slides. Two were used as positive controls and one as a negative control. The optimization process involved staining two slides with varying antibody concentrations (low and high), while the third slide served as a negative control with only secondary antibody incubation. This systematic approach to antibody optimization revealed optimal cytoplasmic staining at a 1:500 dilution, with the negative control slide showing no detectable signal. Immunohistochemical (IHC) analysis was conducted following previously reported methodologies [29]. The TMA slides were deparaffinized in two xylene series and rehydrated through a descending ethanol series. Antigen retrieval was achieved by heating the slides in ethylenediaminetetraacetic acid (EDTA) buffer at pH 8.0 for 25 min, then cooling them to rt. To block the endogenous peroxidase activity, the sections were treated with a 3% H_2_O_2_ solution. After washing, the slides were incubated overnight at 4 °C with rabbit anti-SMYD2 polyclonal antibodies (Proteintech, Rosemont, IL, USA). The antigen–antibody complexes were visualized using the VECTASTAIN Elite ABC HRP Kit (Vector Laboratories, Burlingame, CA, USA). Tissue sections were counterstained with hematoxylin and mounted with a permanent mounting medium. Protein signal detection was performed using a Nikon Eclipse 80i light microscope (Nikon Instruments, Melville, NY, USA). The immunohistochemical score (IHS) was determined by evaluating signal intensity, scored from 0 to 3, and the distribution proportion, scored from 1 to 5, resulting in a maximum possible score of 8 [29]; this scoring system provided a quantitative measure of protein expression, facilitating the comparison of SMYD2 levels across different tissue samples.

### 2.7. Western Blot Analysis

About 1 × 10^6^ NCI-H660 cells and tumor tissues were processed as previously reported [25,26,27,28,29]. Protein bands were visualized using the Chemi-Doc XRS chemiluminescent gel imaging system and analyzed with Image Lab software (BioRad v5.2.1, Hercules, CA, USA, Supplementary Table S3). β-tubulin was used as a loading control. All experiments were performed in triplicate, and representative images are presented in the Section 3 figures.

### 2.8. RNA Extraction

Excised tumors were snap-frozen and preserved in −80 °C until ready to process. Around 50 mg of tumor tissue was weighed and placed in 1 mL of Trizol (Catalogue #15596026, ThermoFisher, Waltham, MA, USA) in an RNase-free Eppendorf tube for 30 min. To ensure complete lysis, tumors were homogenized using a tissue homogenizer at a medium speed for 30 s. This was repeated 3×, with a 1-min difference period on an ice bath between each round until the solution was uniform. Phase separation, RNA precipitation, RNA washing, and resuspension were performed according to the manufacturer’s protocol. RNA was quantified using Nano Drop (ThermoFisher, Waltham, MA, USA) and preserved at −80 °C for subsequent analysis.

### 2.9. Quantitative PCR (qPCR) Analysis

The cDNA was synthesized using a Bio-Rad iScript cDNA synthesis kit (Bio-Rad catalogue #1708891) according to the manufacturer’s protocol. The qPCR was performed on a CFX96 real-time system (Bio-Rad) using the iTaq universal SYBR green super mix (Catalogue #1725121). Primers were designed and synthesized using the prime-quest tool from Integrated DNA Technologies (IDT, Coralville, IA, USA). EZH2 F 5′-GAC CTC TGT CTT ACT TGT GGA GC-3′ EZH2 R 5′–CGT CAG ATG GTG CCA GCA ATA G-3′ SYMD2 F 5′ AAG GCA GAA GCC ATC CGA GAC A-3′ SYMD2 R 5′ TCA TCT TCT CCT GGC TGA GCT C-3′ c-MET F 5′ TGC ACA GTT GGT CCT GCC ATG A-3’ C-Met R 5’ CAG CCA TAG GAC CGT ATT TCG G–3′ β-actin F 5′ GCA CCA CAC CTT CTA CAA TGA-3′. β-actin R 5′ GTC ATC TTC TCG CGG TTG GC–3′. β-actin served as a loading control. Results were normalized to the control untreated group, and fold-change was quantified using the ΔΔCT method. Experiments were conducted in duplicates and replicated 3 times independently.

### 2.10. Animal Models and Treatments

Male athymic nude mice (Foxn1^nu^/Foxn^1+^, 4–5 weeks old) were obtained from Envigo (Indianapolis, IN, USA, Supplementary Table S4) and housed at the University of Louisiana at Monroe (ULM) vivarium. Mice were acclimated for one week under sterile, clean room conditions in ventilated filter-top cages with AlphaDri bedding. The vivarium maintained a controlled environment at 24 ± 2 °C, with 50–65% relative humidity and a 12 h light/dark cycle. Cages were cleaned and bedding changed twice weekly to minimize stress and maintain hygiene. Mice had access to sterile water and pelleted rodent chow containing 5% fat (Cat #7012, Envigo-Teklad, Madison, WI, USA). Animal procedures were conducted in compliance with the NIH guidelines and approved by the ULM Institutional Animal Care and Use Committee (IACUC) under protocol numbers 22-OCT-KES-01 and 23-MAR-KES-02. Clinical health parameters, including food and water consumption, defecation, urination, physical activity, and body weight, were monitored daily throughout this study.

### 2.11. Nude Mouse Xenograft Model

Approximately 2 × 10^6^ NCI-H660-Luc cells in 100 µL of Matrigel were subcutaneously injected into the right back flank of each male nude mouse. Tumor progression was monitored daily, and the tumor volume (V) was calculated using the formula V = (L/2) × W^2^, where L is tumor length, and W is tumor width. The experimental design included both tumor progression and recurrence phases, with primary tumors surgically excised before recurrence phase initiation. Mice continued to receive either daily OC or placebo control treatments after primary tumor excision surgery, with close monitoring to ensure welfare and human care. When each mouse developed a palpable tumor of ~50 mm^3^ at the xenografting site, they were randomized into two groups (*n* = 5 each): (i) placebo control; and (ii) 10 mg/kg daily oral OC treatments started and continued daily for 47 days. Oral administration used 2-millimeter-diameter gavage tubes with a stainless-steel bite protector, gauge 23, 3.81 cm long. Mice were then anesthetized with isoflurane, and primary tumors surgically excised under aseptic conditions. Wounds were clipped and monitored for contamination-free healing. Mice were kept under strict clinical observation for 24 h post-surgery. The next day, each group resumed their respective oral treatments: placebo control and daily oral OC 10 mg/kg for an additional 45 days. Tumor dimensions, measured by digital caliper and tumor volumes, were calculated. Mice were anesthetized with isoflurane at the end of the study and finally euthanized. Tumors and organs were excised, weighed, and stored at −80 °C for subsequent total protein extraction and Western blot analyses.

### 2.12. LuCaP 93 PDX Transplanted in an NSG Mice Model

The LuCaP 93 is a PDX model of t-NEPC, which was acquired from the NCI Patient-Derived Xenograft (PDX) Models Repository (PDMR, Frederick, MD, USA). The t-NEPC LuCaP 93 PDX tumors were utilized to evaluate OC treatment effects after transplantation in NOD scid gamma (NSG) mice purchased from Jackson Laboratories-MMRRC (Stock# 005557, Bar Harbor, ME, USA). A small LuCaP 93 PDX fragment, 2–3 mm in diameter or 20–30 mm^3^, was subcutaneously aseptically transplanted into the right back flank of each NSG mouse. When the transplanted tumors reached a palpable size of 50 mm^3^, the mice were randomized into two groups (*n* = 5, each). The first group was treated with a placebo control, dephenolized EVOO (dpEVOO), an EVOO from which all phenolics are extracted, as previously described [28]. The second group received daily oral OC at 10 mg/kg. Treatments continued until the placebo control tumors reached a volume of 300–400 mm^3^, after nearly 49 days. Tumor measurements and volume calculations were performed as previously described for the nude mice xenograft model. Mice were anesthetized with isoflurane and euthanized at the end of the study. Tumors and organs were excised, weighed, and stored at −80 °C for subsequent analyses.

### 2.13. RNA Sequencing and Data Analysis

RNA sequencing was conducted at the University of Kansas Medical Center Genomics Core with a strand-specific, 100-cycle paired-end configuration using the Illumina NovaSeq 6000 platform (Illumina, San Diego, CA, USA). The NCI-H660-Luc and LuCaP 93 tumor samples, treated with OC and placebo control, were multiplexed across two lanes of a flow cell, yielding between 40.04 and 48.71 million reads per sample. The read quality was evaluated with FastQC software v0.12.0 [30], revealing an average Phred quality score > 30 across all samples. No adapter sequences requiring trimming were detected during quality control. Reads were aligned to a combined human (GRCh38) and mouse (GRCm39) reference genome using STAR software (version 2.7.11b2) [31]. On average, 91% of reads mapped to the combined genome, with 80.4% of those reads mapping uniquely, resulting in 36.0 to 42.9 million uniquely mapped reads per sample. Transcript abundance was quantified using FeatureCounts v2.07 [32], and differential gene expression analysis was performed with DESeq2 v1.48.2 [33]. The *p*-values were adjusted for multiple testing using the Benjamini and Hochberg method [34] to control the false discovery rate (FDR) for each gene.

### 2.14. Pathway Enrichment Analysis (PEA)

Statistical analysis and the interpretation of differentially expressed genes (DEGs) were conducted using R software (version 4.4.2). Pathway enrichment analysis (PEA) was performed through gene set enrichment analysis (GSEA) using gene sets from the Gene Ontology (GO) databases [35]. The Bioconductor packages ClusterProfiler v4.12.6, enrichR v1.24.4, and DOSE v4.0.0 were utilized for hierarchical clustering and functional enrichment analysis of the DEGs [36]. Gene Ontology (GO) was applied in PEA to investigate the affected biological processes, cellular components, and molecular functions associated with the DEGs [35]. Data visualizations were generated using Tidyverse packages v2.0.0 [37].

### 2.15. Protein–Protein Interactions

The downregulated DEGs were uploaded and analyzed using STRING v12.0, with a confidence threshold set at 0.4. This confidence score ranges from 0 to 1, with 1 representing the highest confidence. It reflects the likelihood that STRING deems an interaction to be valid, based on evidence from the genomic context, gene co-expression, experimental interactions, molecular pathways, automated text mining, and curated data.

### 2.16. Statistics

Data analysis was performed using GraphPad Prism software, version 8.4.3 (La Jolla, CA, USA). The results are expressed as mean ± standard deviation (SD) for continuous variables. Statistical analyses were performed using unpaired *t*-tests and one-way ANOVA. Statistical significance was determined with p-values of * *p* < 0.05, ** *p* < 0.01, *** *p* < 0.001, and **** *p* < 0.0001.

## 3. Results

### 3.1. SMYD2 Is a Contributing Factor for NCI-H660 De Novo NEPC Cell Survival

**Figure 1:**
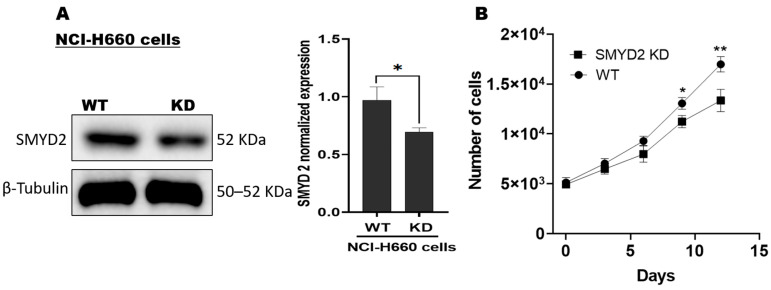
Effect of SMYD2 knockdown on NCI-H660 cell viability: (**A**) Successful SMYD2 knockdown evidenced by comparing the Western blot of the parent cells versus SMYD2-KD NCI-H660 cell lysates. (**B**) Comparison of the viability of the parent NCI-H660 cells versus NCI-H660-KD cells in cell proliferation assay over 15 days. Vertical bars represent the normalized SMYD2 expression in NCI-H660-KD cells relative to parent control cells. Data are presented as mean ± SD (*n* = 3); Student’s *t*-test; * *p* < 0.05, ** *p* < 0.01. WT: Parent cells; KD: Knockdown.

The SMYD2-specific shRNA plasmid–lipofectamine was used to knock down (KD) SMYD2 in NCI-H660 de novo NEPC cells, NCI-H660-KD. SMYD2 knockdown efficiency was confirmed by Western blotting, which averaged a 33.1% reduction in SMYD2 protein levels in NCI-H660-KD cells compared to the parent cells (Figure 1A and Supplementary Figure S3). Subsequently, the impact of SMYD2 KD on cell viability was evaluated. Over a span of 15 days post-transfection, the NCI-H660-KD cells averaged a 21.4% reduction in cellular growth. Considering that the doubling time for the control NCI-H660 cells is ~100 h [38], the results emphasize the essential role of SMYD2 in the survival of the de novo NEPC NCI-H660 cells (Figure 1B).

### 3.2. OC Attenuated NCI-H660 Cell Proliferation and Suppressed SMYD2 Expression

**Figure 2:**
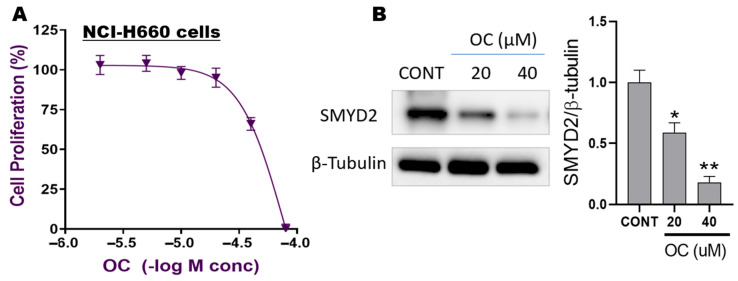
Effects of OC treatments on NCI-H660 cell viability and SMYD2 expression level: (**A**) Antiproliferative effects of OC on the de novo NEPC NCI-H660 cells. Dose–response curve showing the inhibition of NCI-H660 cell proliferation at different OC concentrations. (**B**) Western blot analysis of SMYD2 expression in NCI-H660 cells treated with VC, 20 μM OC, and 40 μM OC. Representative Western blots showing the dose-dependent reduction in SMYD2 expression. Densitometric quantification of SMYD2 level was performed for all blots, with each experiment conducted in triplicate. The integrated optical density of SMYD2 bands was normalized to β-tubulin loading control. Bar graphs represent the mean relative protein expression of SMYD2 as a percentage of the vehicle-treated control (± SD, *n* = 3). Statistical significance was determined using unpaired *t*-tests, with * *p* < 0.05 and ** *p* < 0.01 indicating significant differences compared to the vehicle-treated control.

OC effectively suppressed the proliferation of the de novo NCI-H660 NEPC cells in dose- and time-dependent manners. The calculated OC IC_50_ was 32.4 μM, suggesting a moderate in vitro antiproliferative activity (Figure 2A). To investigate the effect of OC on SMYD2 expression, NCI-H660 cells were treated with a subtoxic dose of 20 μM and a dose slightly higher than the IC_50_, 40 μM. Protein lysates were analyzed by Western blotting to assess changes in SMYD2 expression. OC dose-dependently reduced SMYD2 expression versus the vehicle-treated control (Figure 2B). Specifically, 20 μM OC reduced the SMYD2 level by ~50%, while the 40 μM OC treatment resulted in an 80% reduction in SMYD2 expression. Densitometric analysis of the Western blots was normalized to the β-tubulin loading control (Figure 2B).

### 3.3. Differential SMYD2 Expression in PCa Versus Non-Tumorigenic Prostate Tissues

**Figure 3:**
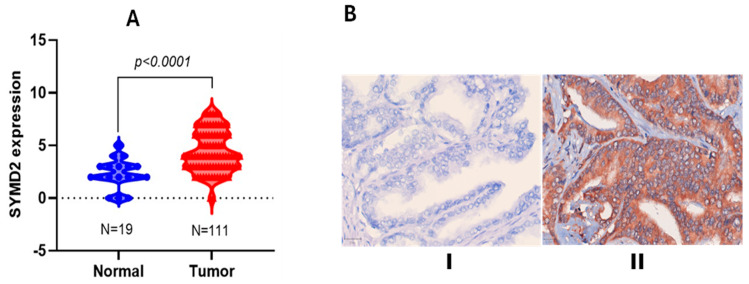
TMA expression of SMYD2 in PCa clinical tissues: (**A**) Comparison of SMYD2 expression in normal prostate versus PCa tissues expressed as immunohistochemical score (IHS). Unpaired *t*-tests calculated data significance at *p* < 0.0001. (**B**) Comparison of the SMYD2 expression IHS in normal prostate tissues (**I**) versus tissues collected from PCa patients with Gleason scores > 7 (**II**) at 400×.

SMYD2 expression displayed significant upregulation in PCa tissues compared to benign prostate tissues [39]. Clinical data for both PCa and non-tumorigenic patient tissues were reviewed and considered during this process. SMYD2 expression was evaluated by IHC in a TMA slide comprising 64 cases/192 PCa tissue cores and 6 normal prostate cases/18 tissue cores. SMYD2 expression was significantly elevated in PCa tissues (*p* < 0.0001) with higher IHS versus normal prostate tissue cores (Figure 3A). SMYD2 expression was lower in normal prostate tissues compared with PCa patients with Gleason scores > 7, indicating SMYD2 overexpression in advanced-grade PCa (Figure 3B). Thus, SMYD2 is critical for PCa pathogenesis, especially in the advanced stages, and therefore it can be considered a clinically valid molecular target.

### 3.4. Daily Oral OC Treatments Suppressed the De Novo NEPC Progression and Recurrence and t-NEPC PDX Progression

**Figure 4:**
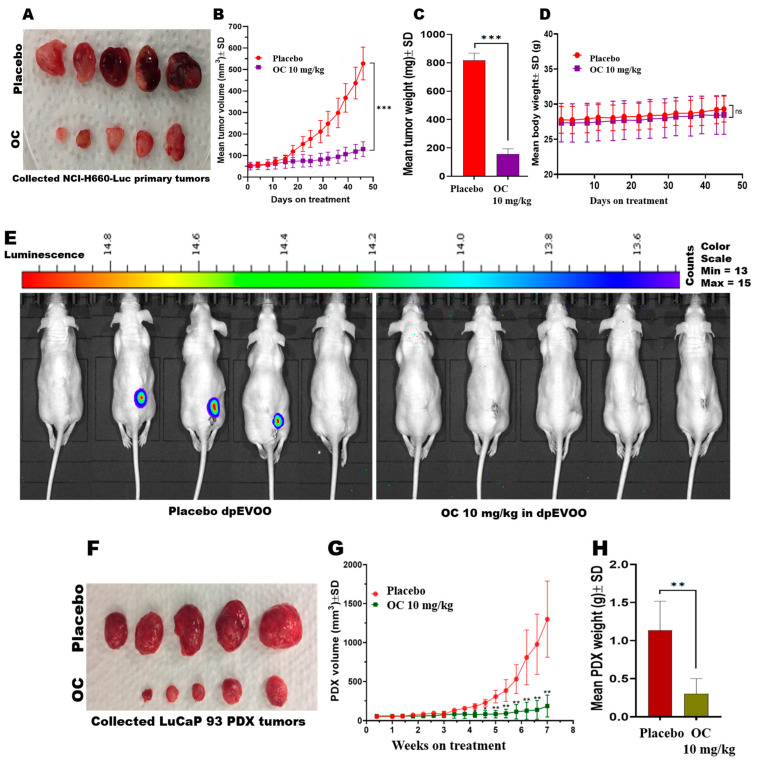
Effects of OC on de novo NEPC NCI-H660-Luc tumor progression and locoregional recurrence, and t-NEPC LuCaP 93 PDX tumor progression: (**A**) Photographs comparing the collected OC versus placebo control-treated NCI-H660-Luc primary tumors after surgical excision. (**B**) NCI-H660-Luc tumor volume monitoring over the course of the study. (**C**) Comparison of OC versus placebo control-treated NCI-H660-Luc primary tumor weight. (**D**) Mean NCI-H660-Luc tumor-bearing nude mice body weight monitoring over the course of the study. (**E**) Whole-body-weight imaging of nude mice at the end of NCI-H660-Luc tumor recurrence experiment comparing OC versus placebo control-treatment effects on locoregional recurrence. (**F**) Photographs comparing the collected OC versus placebo control-treated LuCaP 93 PDX tumors in NSG at the end of the study after 7 dosing weeks. (**G**) Comparison of OC versus placebo control-treated LuCaP 93 PDX tumor volume over the course of the study. (**H**) Comparison of OC versus placebo control-treated LuCaP 93 PDX tumor weight at the end of the study. Data are presented as mean ± SD. * *p* < 0.05, ** *p* < 0.01, *** *p* < 0.001, ns: not significant.

Daily 10 mg/kg oral OC use for 47 days significantly reduced NCI-H660-Luc tumor burden in the nude mice xenograft model, achieving 80% and 83.7% tumor volume and weight reductions, respectively, compared to the placebo dp-EVOO group (Figure 4A–C and Supplementary Figure S1). Importantly, no significant differences in the study animals’ body weights were observed between the OC and placebo control-treated groups throughout this study. This indicated that OC treatments were well tolerated and showed a preliminary safety profile (Figure 4D). Locoregional tumor recurrence was observed in 3 out of 5 mice in the placebo group, whereas OC treatments completely prevented any locoregional recurrence by the end of the study, 45 days after primary tumor excision surgery (Figure 4E). Morphologically, OC-treated primary tumors revealed a significant vascularity reduction versus the placebo group (Figure 4A). The results highlight that OC is effective in the suppression of de novo NEPC progression and locoregional recurrence.

OC treatments’ suppressive efficacy against the progression of the t-NEPC LuCaP 93 PDX transplanted in the NSG mice was evaluated. Daily oral OC 10 mg/kg treatments for 49 days suppressed 85.7% and 82.6% of the LuCaP 93 tumors’ volume and weight, respectively, compared to the placebo control (Figure 4F–H and Supplementary Figure S2). LuCaP 93 tumors revealed markedly reduced morphological vascularity in the OC-treated group relative to the placebo control (Figure 4F). The results demonstrate OC’s suppressive effects against t-NEPC LuCaP 93 PDX progression.

### 3.5. OC Downregulates ROR2-ASCL1, SMYD2-EZH2, and c-MET in NCI-H660-Luc Cell Primary Tumors and LuCaP 93 PDX Model

**Figure 5:**
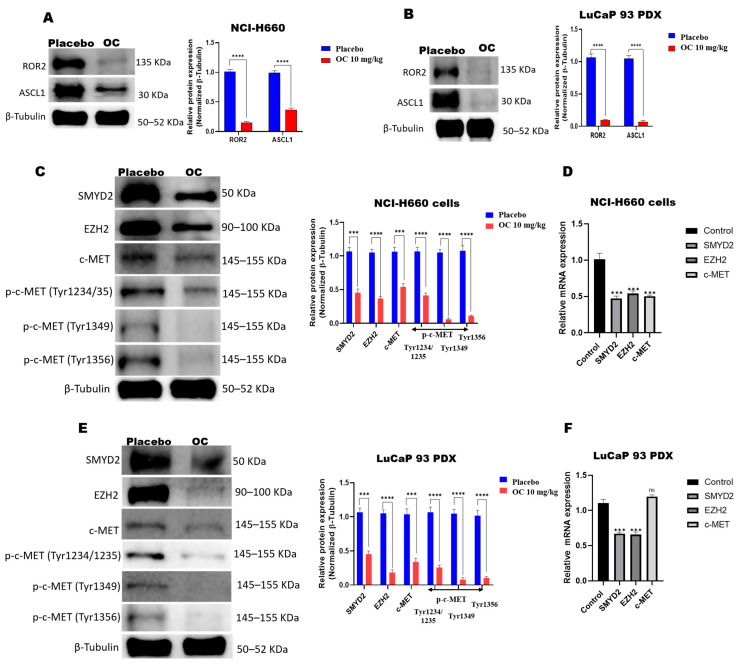
Molecular effects of OC on NCI-H660-Luc and LuCaP 93 PDX tumors. Tumor lysates were prepared by pooling tissues from three randomly selected mouse tumors (out of five per group) for each analysis. Western blot analysis showing the effects of OC treatment compared to placebo on (**A**) ROR2 and ASCL1 expression levels in NCI-H660-Luc tumors and (**B**) ROR2 and ASCL1 expression levels in LuCaP 93 PDX tumors. (**C**) Western blot comparison of OC treatment effects on SMYD2, EZH2, c-MET, p-c-MET Tyr1234/1235, p-c-MET Tyr1349, and p-c-MET Tyr1356 expression levels in NCI-H660 primary tumors versus the placebo control. Densitometric analysis quantified protein levels, with all blots performed in triplicate. The integrated optical density of each band was normalized to β-tubulin loading control. Bar graphs next to the Western blot images display the normalized optical density for each protein. Data are expressed as mean ± SEM (*n* = 3). *** *p* < 0.001, **** *p* < 0.0001 versus placebo control analyzed by one-way ANOVA. (**D**) qPCR analysis indicated a significant reduction in mRNA expression levels of SMYD2, EZH2, and total c-MET in OC-treated NCI-H660 primary tumors compared to placebo controls. Data are presented as mean ± SEM *(n* = 3). *** *p* <0.001 versus placebo control, analyzed by unpaired *t*-tests. (**E**) Western blot comparison of OC treatment effects on SMYD2, EZH2, c-MET, p-c-MET Tyr1234/1235, p-c-MET Tyr1349, and Tyr1356 expression levels in LuCaP 93 PDX tumors versus placebo controls. Densitometric analysis of the blots was performed in triplicate. The integrated optical density of each band was normalized to β-tubulin loading control. The bar graphs next to the blots represent the normalized optical density for each protein. Data are presented as mean ± SEM (*n* = 3). Statistical significance is indicated by *** *p* < 0.001, **** *p* <0.0001, analyzed by one-way ANOVA. (**F**) qPCR analysis revealed significant reduction in mRNA expression levels of SMYD2, EZH2, and increase in total c-MET in OC-treated LuCaP 93 PDX tumors versus placebo control. Data are expressed as mean ± SEM (*n* = 3). Statistical significance indicated by *** *p* <0.001; ns is non-significant, analyzed by unpaired *t*-test.

This study assessed the effect of OC treatments on the expression levels of ROR2 and its downstream substrate ASCL1, lysine methyltransferase SMYD2 and its downstream target EZH2, as well as c-MET and its different phosphorylated forms (Try1234/1235, 1349, and 1356) in both NCI-H660-Luc and LuCaP 93 PDX tumors. Protein expression and mRNA expression levels were evaluated by Western blotting and quantitative polymerase chain reaction analyses (qPCR), respectively. In NCI-H660-Luc tumors, OC significantly reduced the expression of ROR2 (85%) and ASCL1 (64%) versus control tumors (Figure 5A and Supplementary Figure S4). A similar trend was observed in the LuCaP 93 PDX model, where OC reduced 91% of ROR2 and 93% of ASCL1 expression (Figure 5B and Supplementary Figure S5). OC treatments induced a remarkable 55% reduction in total SMYD2 level in NCI-H660-Luc tumors. Additionally, EZH2 expression was also markedly attenuated by OC, with a 64% reduction observed. OC treatments significantly suppressed total c-MET level by 46% and its activated/phosphorylated tyrosine residues 1234/1235, 1349, and 1356 by 74%, 94%, and 89%, respectively (Figure 5C). The qPCR analyses further corroborated these findings at the mRNA level. qPCR results revealed significant suppression of SMYD2 mRNA expression by OC treatments, resulting in a 53% reduction in NCI-H660 tumors. OC treatments also reduced the EZH2 mRNA level by 46%, along with total c-MET mRNA expression by 50%, in NCI-H660 tumors (Figure 5D and Supplementary Figure S6).

Similar inhibitory effects are observed in LuCaP 93 PDX tumors. SMYD2 expression level was reduced by 55%, while EZH2 was diminished by 82% following OC treatments (Figure 5E and Supplementary Figure S6). OC downregulated the total c-MET expression level by 63%. Similarly, the OC treatments downregulated the levels of activated/phosphorylated c-MET Tyr1234/1235, 1349, and 1356 by 74%, 92%, and 90%, respectively, in LuCaP 93 PDX tumors (Figure 5E). The qPCR analysis revealed a 33% reduction in the SMYD2 mRNA and a 34% decrease in EZH2 mRNA levels. LuCaP 93 PDX c-MET mRNA expression increased by 20% in the OC-treated group, suggesting potential compensatory mechanisms (Figure 5F). Collectively, the results demonstrate that OC treatments robustly downregulated the ROR2-ASCL1 and SMYD2-EZH2 signaling axes and inhibited the total and activated c-MET signaling in both primary de novo NEPC tumors and t-NEPC PDX models, providing unique mechanistic insights into OC’s therapeutic potential against NEPC.

### 3.6. Pathway Enrichment Analysis and Protein–Protein Interactions of DEGs

**Figure 6:**
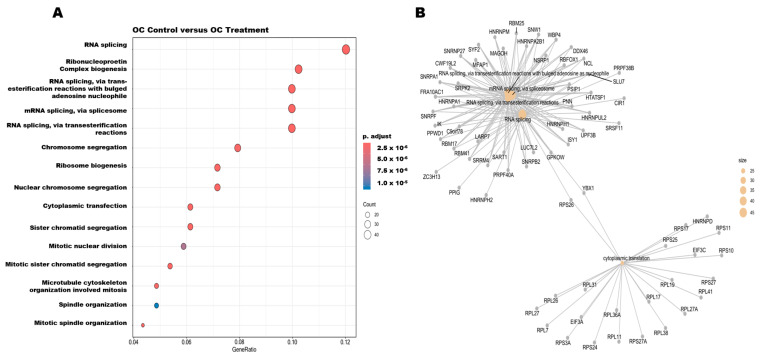

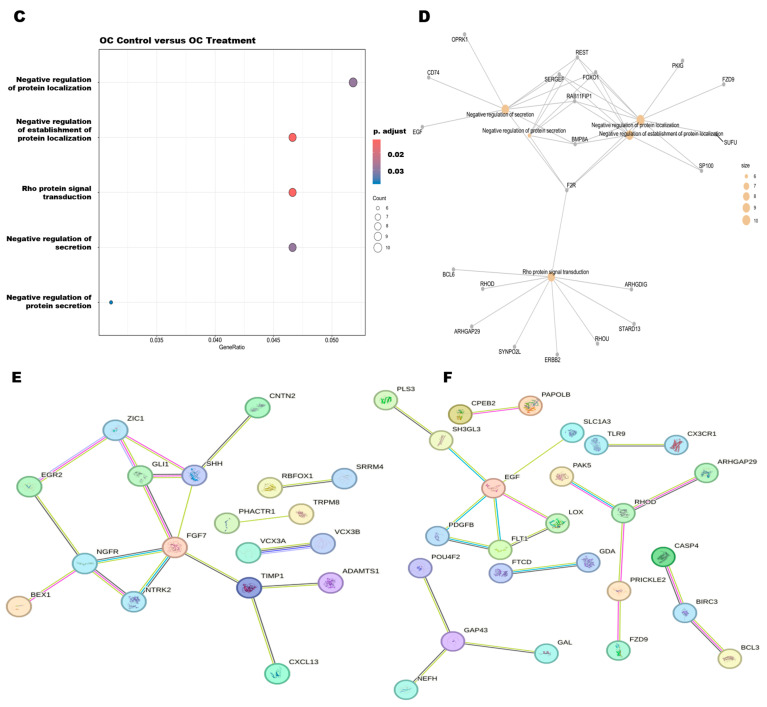
GO enrichment and PPI network analysis of downregulated DEGs in NCI-H660 and LuCaP 93 PDX tumors following OC treatments: (**A**) Dot plot illustrates the proportion of significantly enriched downregulated DEGs associated with distinct biological processes in NCI-H660 tumors. (**B**) Cnet plot visualizing the interaction network of downregulated DEGs mapped to the topmost five significantly enriched biological processes in NCI-H660 tumors. (**C**) Dot plot depicting the ratio of significantly enriched downregulated DEGs in LuCaP 93 PDX tumors, highlighting the affected biological pathways. (**D**) Cnet plot showing the network connectivity of downregulated DEGs linked to topmost five enriched biological processes in LuCaP 93 PDX tumors. DEGs identified using log2FC > 1.5 or log2FC < −1.5 with an adjusted *p*-value < 0.05. (**E**) STRING network analysis of downregulated DEGs in NCI-H660 tumors after OC treatments revealed 53 nodes and 18 edges, with a PPI enrichment *p*-value of 0.000227 (log2FC < −5, adjusted *p*-value < 0.05). (**F**) STRING analysis of downregulated DEGs in LuCaP 93 PDX tumors in response to OC treatments identified 80 nodes and 20 edges, with a PPI enrichment *p*-value of 0.0207 (log2FC < −3, adjusted *p*-value < 0.05).

RNA sequencing analysis revealed a total of 423 differentially expressed genes (DEGs) when comparing OC treatments versus the placebo control in NCI-H660 tumors, including 41 genes upregulated and 382 genes downregulated, using a cutoff criterion of log2FC > 1.5 or log2FC < −1.5 and an adjusted *p*-value < 0.05. Gene Ontology (GO) enrichment analysis was performed for downregulated genes using clusterProfiler and enrichR, using a significance threshold set at a *p*-adjusted value < 0.05 and a gene count > 5. The *p*-values were adjusted via the Benjamini–Hochberg procedure to limit the false discovery rate. NCI-H660 tumors showed 69 of 382 downregulated genes mapped to 15 significantly enriched GO pathways (Figure 6A). These genes were predominantly associated with RNA splicing and translation processes (Figure 6B). The five topmost significantly enriched pathways included RNA splicing via transesterification reactions with bulged adenosine as a nucleophile, mRNA splicing via the spliceosome, RNA splicing via transesterification reactions, RNA splicing, and cytoplasmic translation. The LuCaP 93 PDX tumors showed 21 out of 201 downregulated genes enriched across five significant GO pathways (Figure 6C). These genes are primarily linked to negative regulatory processes (Figure 6D), including negative regulation of protein localization, negative regulation of establishment of protein localization, Rho protein signal transduction, and negative regulation of protein secretion in LuCaP 93 PDX tumors.

To investigate functional connections among downregulated DEGs, protein–protein interaction (PPI) networks were constructed using STRING. Analysis of OC treatments on NCI-H660 tumor PPIs, using a cutoff criterion of log2FC < −5 and adjusted *p*-value < 0.05, identified 53 nodes and 18 edges, with an expected number of edges at 7. The PPI enrichment *p*-value was 0.000227, with a confidence cutoff of 0.4, indicating that the proteins had significantly more interactions than would be randomly expected. This enrichment suggests that affected proteins are biologically connected and may contribute to shared regulatory pathways. Further analysis identified key genes forming strong connections, including SHH, ZIC1, GLI1, FGF7, NTRK2, NGFR, BEX1, EGR2, CNTN2, TIMP1, CXCL13, and ADAMTS1. The PPI analysis elucidated the interconnected nature of downregulated genes, with key hubs such as SHH and ZIC1 in NCI-H660-Luc tumors implicated in developmental and neurogenesis-related pathways (Figure 6E). Analysis of OC treatments on LuCaP 93 PDX tumors, with a cutoff of log2FC < −3 and adjusted *p*-value < 0.05, revealed 80 nodes and 20 edges, with an expected number of edges at 12. The PPI enrichment *p*-value was 0.0207, with a confidence cutoff of 0.4, indicating significant biological connectivity among affected proteins. The topmost three major interaction hubs identified were as follows: firstly, the EGF-centered, including EGF, LOX, FLT1, PDGFB, SLC1A3, SH3GL3, and PLS3; secondly, the RHOD-centered, including RHOD, ARHGAP29, PAK5, PRICKLE2, and FZD9; and lastly, the GAP43-centered, including GAP43, GAL, NEFH, and POU4F2. Identification of EGF, RHOD, and GAP43 interaction central nodes suggests the disruption of the growth factor signaling, cytoskeletal dynamics, and neuronal differentiation, which could collectively impair tumor progression and metastasis (Figure 6F).

## 4. Discussion

Despite the therapeutic advances, most PCa patients inevitably develop resistance to most current APIs, especially with extended use [1,2,3]. In CRPC, AR signaling persists through various mechanisms, including AR gene mutations, amplifications, splice variants, and constitutive ligand-independent activation [4,5]. While the overall survival (OS) for t-NEPC patients ranges from 7 to 20 months, de novo NEPC patients have a shorter OS. Both NEPC subtypes exhibit similarly poor outcomes [7]. NEPC is a challenging PCa phenotype with aggressive behavior, poor prognosis, and limited therapeutic options; thus, identifying novel therapeutics to control NEPC is a high therapeutic priority.

ROR2 upregulation plays oncogenic roles in renal cell carcinoma, osteosarcoma, breast cancer, and melanoma [10,11]. ROR2 is the topmost upregulated RTK after the AR pathway inhibition by androgen pathway inhibitors (APIs) [10,11]. ROR2 is minimally expressed in adult normal tissues but is usually upregulated following API use in PCa patients, promoting the neuroendocrine (NE) phenotype [10,11]. The interplay between ROR2 and ASCL1 is critical for CSPC lineage plasticity and therapeutic resistance. ASCL1 functions as a master transcription factor essential for NE differentiation. The ROR2–ASCL1 axis is validated as a molecular circuit where therapeutic pressure suppresses AR signaling, leading to ROR2 induction and downstream ASCL1-mediated reprogramming of the tumor lineage.

SMYD2 plays a crucial role in tumor progression and therapeutic resistance through its involvement in AR signaling, tumor aggressiveness, and metastasis [14]. SMYD2 modulates CRPC cell resistance to enzalutamide by enhancing the AR promoter methylation, AR phosphorylation and ubiquitination, and proteasome degradation [17]. SMYD2 enhances methylation and phosphorylation of AR, contributing to PCa signaling loss and promoting lineage reprogramming [14,17]. Aberrant dysregulation of SMYD2 is associated with tumor aggressiveness and resistance to conventional therapies, justifying its therapeutic potential for NEPC. Current SMYD2 inhibitors demonstrated high in vitro potency and selectivity but showed limited in vivo antitumor efficacy, underscoring the need for developing new SMYD2-targeting leads [14,17,26,40]. SMYD2 knockdown in NCI-H660 cells led to a marked decrease in viability, underscoring its contribution to NEPC pathogenesis. TMA results displayed the clinical importance of SMYD2 based on its high expression levels in advanced PCa cases with a Gleason score > 7 versus normal prostate tissues [26].

Overexpression of EZH2 is observed in NEPC, unlike CRPC, localized PCa, and benign prostate tissues. Targeting EZH2 restores AR expression, re-sensitizes NEPC to AR-targeted therapies, and reverses NEPC lineage plasticity [8]. EZH2 promotes NEPC by upregulating NE drivers like ASCL1 [41,42]. EZH2 enhances c-MET signaling by repressing miR-141/200a, facilitating API’s resistance and metastasis [43,44,45]. The results indicate that OC suppression of ROR2, ASCL1, and EZH2 was more effective in the LuCaP 93 PDX versus NCI-H660 tumors, suggesting its improved efficacy in the more prevalent t-NEPC. c-MET possesses a unique MET-Binding Domain (MBD) hub, intended for diverse PPI with protein substrates and subsequent activation of several downstream cascades [27]. Concurrently, OC effectively suppressed c-MET phosphorylation at tyrosine residues 1234/1235, 1349, and 1356. The MBD Try1349 and 1356 provide a docking platform to recruit the Src homology-2 domain, phosphotyrosine-binding domain, and other critical MBD adapter proteins GAB1, GRB2, phospholipase C, and SRC required for activating cancer motility [27]. The 20% rise in the total c-MET in the LuCaP 93 model and not in the NCI-H660 tumors might not be seriously alarming therapeutic-wise because the active form of this c-MET must be phosphorylated in several tyrosine residues to translate to oncogenic effects towards motility: tyrosines 1234/1235–1349 or proliferation/progression 1234/1235–1356. This is very important because c-MET is the only RTK with a protein–protein interaction (PPI) hub MBD used to activate downstream oncogenic signaling. This study showed OC’s ability to effectively suppress the critical tyrosine residues that drive MBD oncogenic PPIs. The rise in c-MET total level in the OC-treated LuCaP 93 PDX might also be a compensatory mechanism by the LuCaP 93 tumors due to the significant c-MET activation/phosphorylation reduction or potential resistance development, especially if this overexpression was associated with mutation(s). This result might warrant future mechanistic studies, which are outside the scope of this study.

RNA sequencing suggests that OC disrupts RNA processing and protein synthesis, leading to tumor suppression. The enriched pathways included RNA splicing via transesterification reactions and mRNA splicing via the spliceosome. The downregulated genes in the LuCaP 93 PDX model were enriched in pathways related to negative regulation of protein localization and secretion. This may imply that OC treatment interferes with critical cellular signaling processes that regulate protein transport and secretion, which suppresses tumor progression and metastasis. The disruption of protein trafficking can impair various tumor cellular functions, including signal transduction and growth factor receptor activation. The key affected genes in NCI-H660-Luc tumors, including SHH, ZIC1, GLI1, and FGF7, assemble a strong PPI connection network implicated in developmental and neurogenesis-related pathways. Thus, OC may influence neurogenesis-related processes, potentially contributing to suppression of NE differentiation and NEPC progression. PPI analysis revealed EGF-, RHOD-, and GAP43-centered hubs associated with tumor growth factor signaling, cytoskeletal dynamics, and neuronal differentiation in LuCaP93 PDX tumors. Hence, OC may potentially disrupt key NEPC signaling networks, impairing tumor cells proliferation and metastasis.

Epidemiological studies have highlighted that the Mediterranean diet, rich in EVOO, has the ability to reduce the incidence of various cancers [21]. OC is one of the topmost bioactive EVOO phenolics with demonstrated anticancer activities [23,24,25,26,27]. A unique aspect of OC is its possible availability as a promising nutraceutical for both PCa patients and survivors to control disease pathogenesis, minimize disease progression and relapse, and therefore reduce mortality. The effective in vivo 10 mg/kg OC dose used in preclinical mouse models can be translated to a human-equivalent dose of 0.81 mg/kg through multiplication by the 0.081 Km factor ratio for mouse-to-human conversion [46]. When adjusted to a 70 kg average adult human, this equates to a total daily intake of 56.7 mg of OC/day. The standard high-phenolics EVOO usually contains 1000 mg OC/L (1 mg/mL), suggesting possible daily consumption of ~56.7 mL of this EVOO can match the OC dose used in this study. This daily EVOO consumption is realistically within the feasible dietary range, highlighting the translational relevance of OC as a nutraceutical NEPC therapeutic. The acute safety of OC has been comprehensively studied [47]. Oral dosing of 10, 250, and 500 mg/kg OC in male and female Swiss albino mice showed no mortality [47], indicating that the oral OC LD_50_ value is greater than 500 mg/kg, unlike i.p. administration, which showed a 164–524 mg/kg LD_50_ range in these mice. Hence, OC oral safety is far better than the parenteral route. The OECD criteria ranked OC as a class 4 natural product (LD_50_ > 300–2000 mg/kg) [47]. The oral OC LD_50_ of 500 mg/kg in Swiss albino mice can translate to a single-dose toxicity at 35 g of pure OC in humans, with the average 70 kg bodyweight, or 29.2–3500 L of EVOO, assuming an average OC natural occurrence in EVOO ranging from 10 to 1200 mg/L [47]. Chronic 5, 10, and 20 mg/kg OC safety was studied in 5xFAD mice over 3- and 6-month dosing periods in preventive and treatment modes [48]. Overall, OC proved non-toxic except at 20 mg/kg, at which dose hepatotoxicity was recorded in 5xFAD mice [48].

## 5. Conclusions

Collectively, OC daily oral treatments suppressed both NEPC subtypes by targeting the ROR2-ASCL1-SMYD2-EZH2-c-MET axis, indicating a unique molecular profile and justifying potent in vivo potency. OC modulated the critical interconnected oncogenic network that drives lineage plasticity, epigenetic reprogramming, and therapeutic resistance in NEPC. OC is a novel lead intervention useful for the control of NEPC with added translational privilege by availability for immediate use by PCa patients and survivors in clinical trials as a nutraceutical without the need for prior FDA approval.

## Data Availability

All data used to support the findings of this study are available in this publication as figures, tables, or within the Supplementary Materials.

## Supplementary Materials

The following supporting information can be downloaded at https://www.mdpi.com/article/10.3390/nu17243947/s1, Figure S1: Representative images of nude mice xenografted with NCI-H660-Luc cells; Figure S2: Photographs of NSG mice with transplanted t-NEPC LuCaP 93 PDX; Figures S3–S6: Raw Western blotting images. Table S1: RRID of cell line used in the in vitro study model; Table S2: Detailed Research Resource Identifier (RRID) information for antibodies used in the study; Table S3: RRID of software and algorithms used in the study; Table S4: RRID of experimental models: Organisms/strains.
