# Supplementary material for: The Olive Phenolic S–(–)–Oleocanthal as a Novel Intervention for Neuroendocrine Prostate Cancers: Therapeutic and Molecular Insights
Source: Nutrients. 2025 Dec 17;17(24):3947. doi: 10.3390/nu17243947 (PMC12735624; doi:10.3390/nu17243947)
Supplement: Supplementary file 1 [file nutrients-17-03947-s001.zip › nutrients-4024247-supplementary.pdf]

Supplementary Materials

The olive phenolic *S*-(-)-oleocanthal as a novel intervention for neuroendocrine prostate cancers: Therapeutic and molecular insights

Md Towhidul Islam Tarun, Hassan Y. Ebrahim, Dalal Dawud, Zakaria Y. Abd Elmageed, Eva Corey, Khalid A. El Sayed

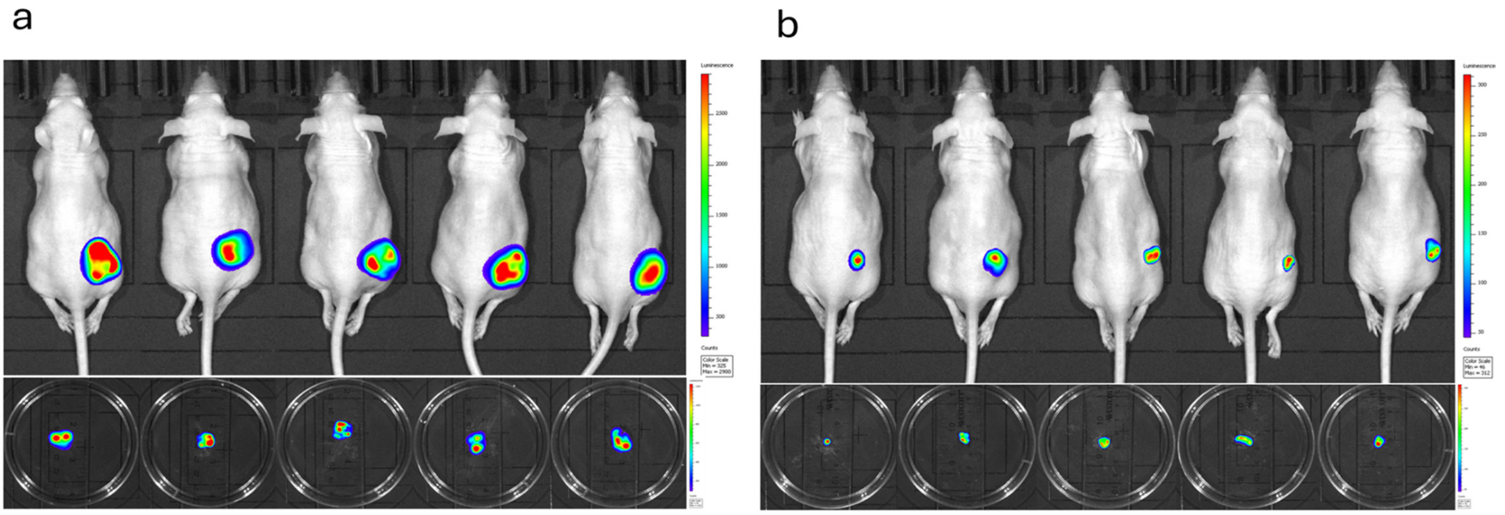

Figure S1. Representative images of nude mice xenografted with NCI-H660-Luc cells, illustrating the potent antitumor effects of oleocanthal prior the primary tumor surgical excision surgery (OC). (a) Placebo group received dephenolized EVOO (dpEVOO). (b) Treatment group received daily oral 10 mg/kg administration of OC formulated in dpEVOO for 45 days in tumor progression study.

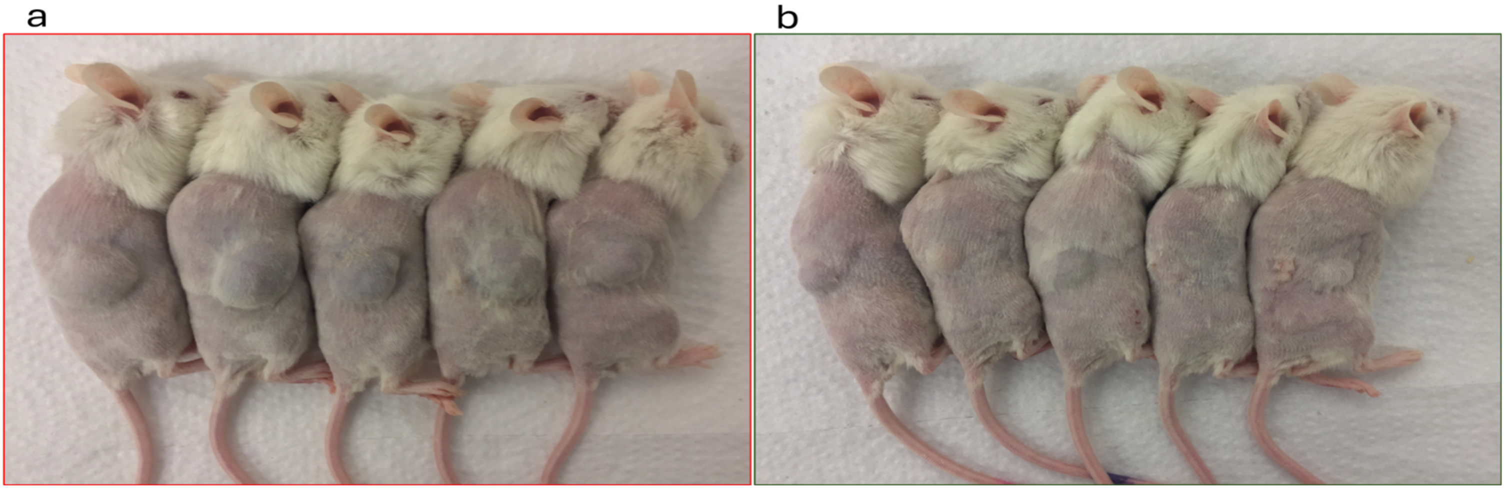

Figure S2. Photographs of NSG mice with transplanted t-NEPC LuCaP 93 PDX showing the exceptional OC antitumor effects. (a) Placebo group received oral dpEVOO. (b) OC-treated group received daily oral dosing of 10 mg/kg in dpEVOO over seven weeks.

SMYD2 KD

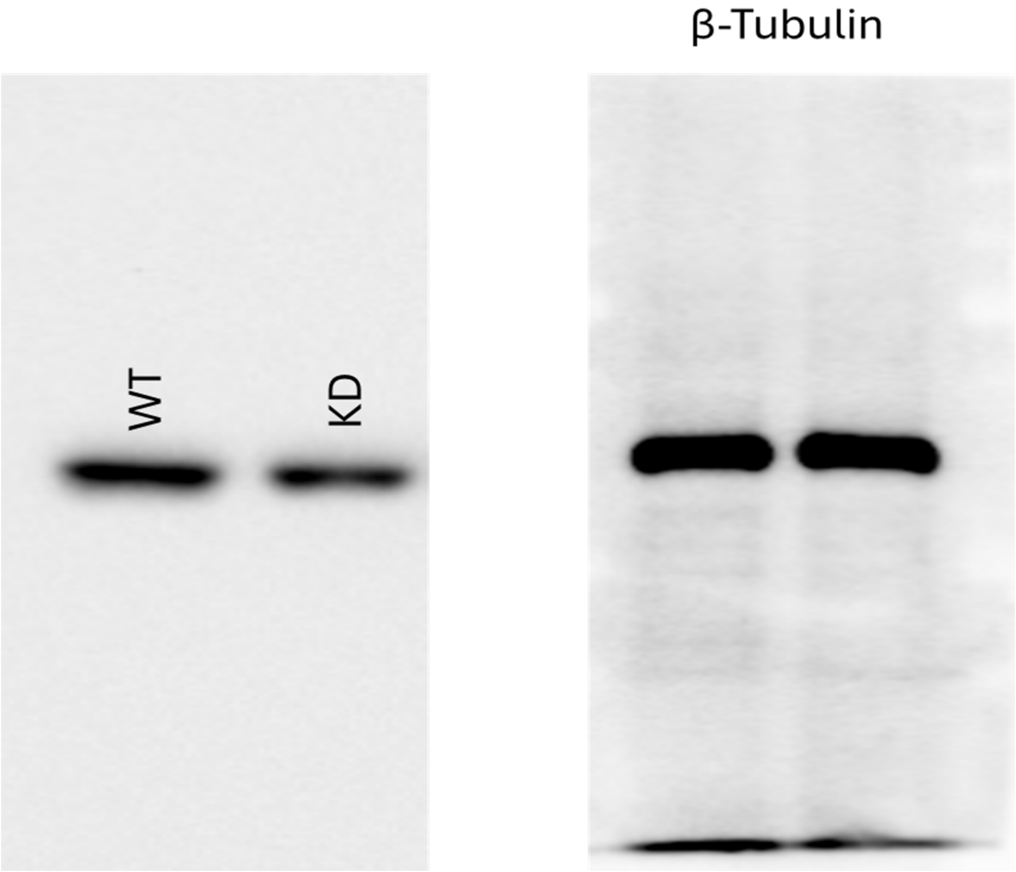

Figure S3. Raw Western blot images validating successful SMYD2 knockdown.

NCI-H660

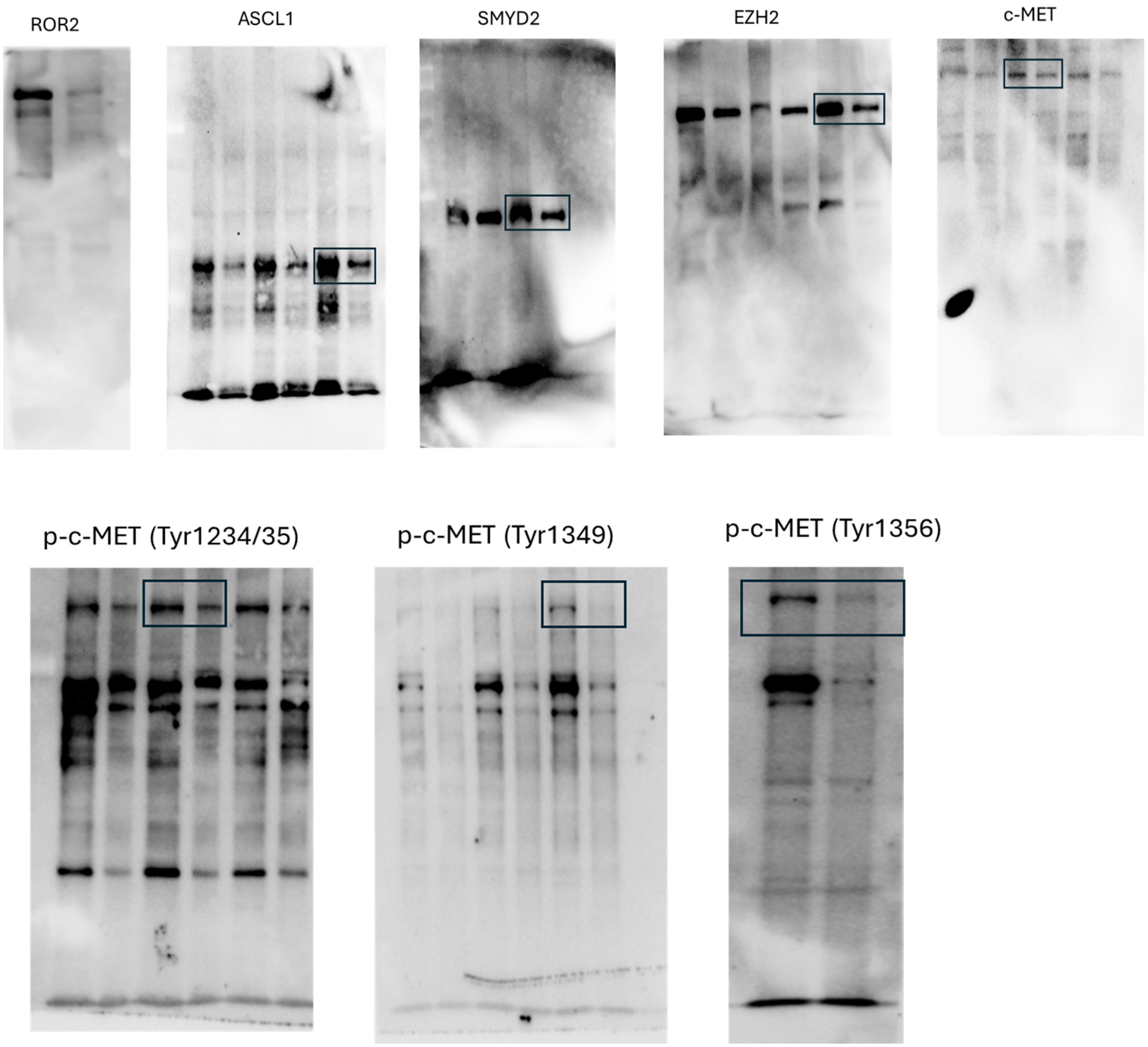

Figure S4. Raw Western blot images of the OC effects on expression levels of ROR2, ASCL1, SMYD2, EZH2, total, and activated c-MET in NCI-H660-Luc tumor tissues.

LuCaP 93 PDX

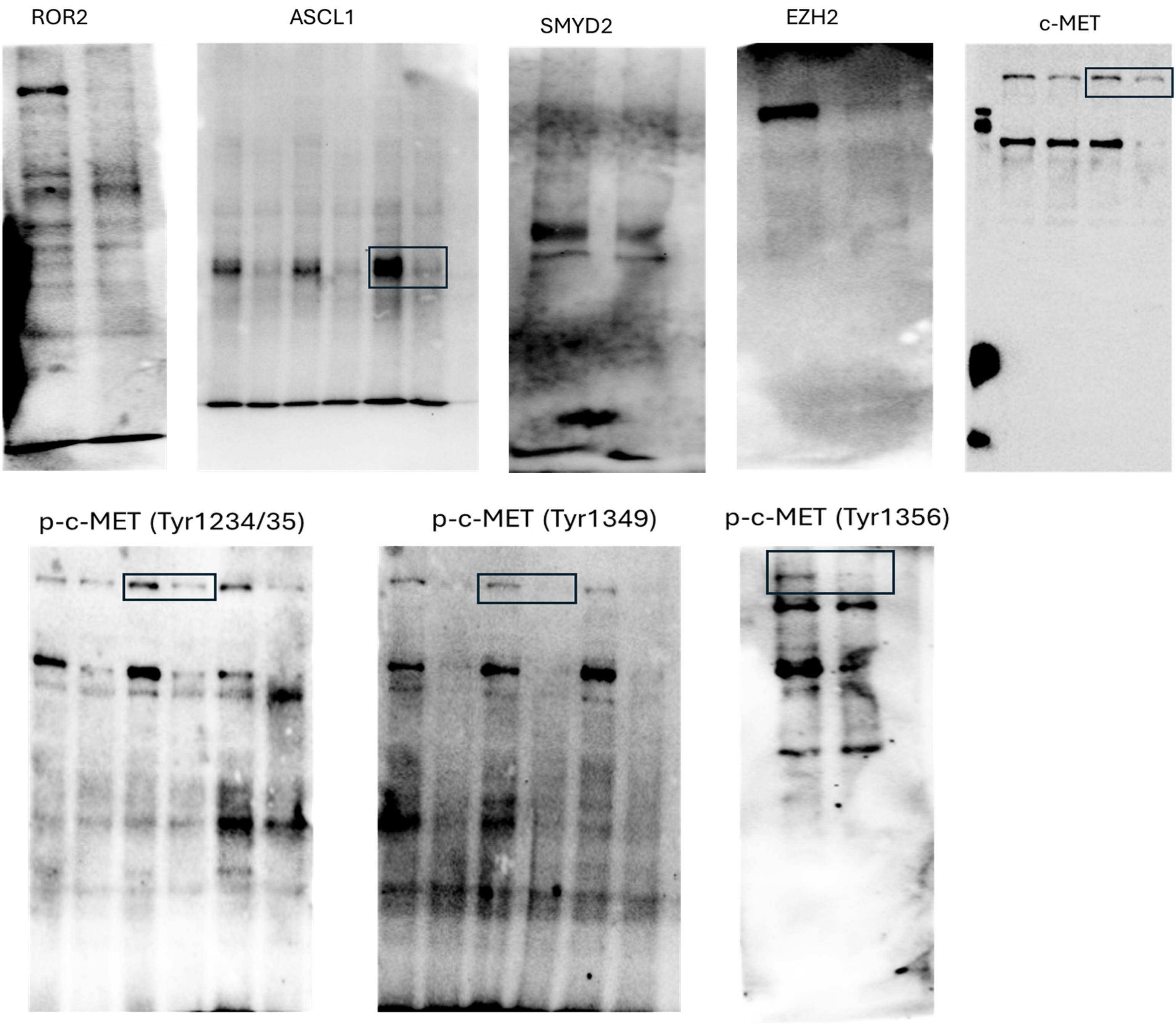

Figure S5. Raw Western blotting images for OC treatments effect on the expression of ROR2, ASCL1, SMYD2, EZH2, and both total and phosphorylated c-MET in LuCaP 93 PDX tumor tissue samples.

β-Tubulin

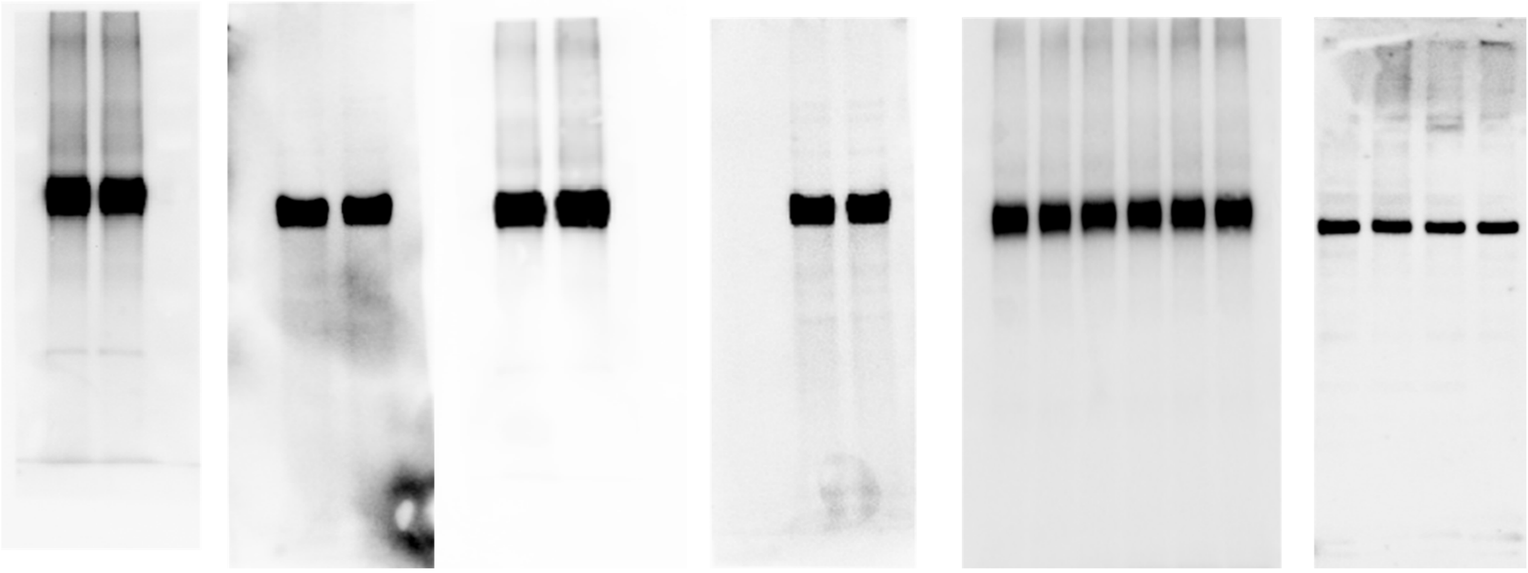

Figure S6. Raw Western blot images showing β-Tubulin used as a loading control in various the experiments.

Supplementary Table S1. RRID of cell line used in the in vitro study model.

Cell Line	RRID
NCI-H660	CVCL_1576

Supplementary Table S2. Detailed Research Resource Identifier (RRID) information for antibodies used in the study.

Antibodies (Catalog/ Ref#)	Source	RRID
ROR2 Rabbit mAb (88639)	Cell Signaling Technology	AB_2800126
ASCL1 Rabbit mAb (10585)	Cell Signaling Technology	NA
SMYD2 Rabbit PolyAb (21290-1-AP)	Proteintech	AB_10733881
EZH2 Rabbit PolyAb (21800-1-AP)	Proteintech	AB_10858790
c-MET Rabbit PolyAb (25869-1-AP)	Proteintech	AB_2880276
Phospho-Met (Tyr1234/Tyr1235) (3077)	Cell Signaling Technology	AB_2143884
Anti-Met (c-Met) (Phospho Tyr1349) (A10883)	Antibodies.com	NA
Anti-Met (Phospho Tyr 1356) (A94043)	Antibodies.com	NA
β-Tubulin (10094-1-AP)	Proteintech	AB_2210695
Anti-rabbit IgG, HRP-linked Antibody (7074S)	Cell Signaling Technology	AB_2099233

Supplementary Table S3. RRID of software and algorithms used in the study.

Software and Algorithms	RRID
PubMed	SCR_004846
FastQC	SCR_014583
Bioconductor	SCR_006442
ClusterProfiler	SCR_016884
enrichR	SCR_001575
STRING	SCR_005223
GraphPad Prism	SCR_002798
Bio-Rad	SCR_008426

Supplementary Table S4. RRID of experimental models: Organisms/strains.

Organisms/Strains	RRID
NU-Foxn^{1nu} athymic nude male mice	IMSR_JAX:000819
NOD scid gamma (NSG) mice	IMSR_JAX:005557
